# Characterizing motor–cognitive associations in Parkinson’s disease using digital assessments

**DOI:** 10.3389/fnagi.2026.1787521

**Published:** 2026-02-23

**Authors:** Avigail Lithwick Algon, William Saban

**Affiliations:** 1 Center for Accessible Neuropsychology, Tel Aviv, Israel; 2 Department of Occupational Therapy, Gray Faculty of Medical and Health Sciences, Tel Aviv University, Tel Aviv, Israel; 3 Sagol School of Neuroscience, Tel Aviv University, Tel Aviv, Israel

**Keywords:** CAN, cognition, motor evaluation, online, Parkinson’s

## Abstract

Traditional motor and cognitive assessments for people with Parkinson’s disease (PD) have long faced challenges related to accessibility, scalability, and geographical diversity when administered in-person. Remote cognitive testing offers the promise of improved accessibility, reduced participant burden, and greater scalability, particularly for individuals with mobility limitations or limited access to specialized care. While remote cognitive assessments are increasingly used in PD research and clinical settings, it remains unclear whether these measures provide an informative index of PD severity. These gaps limit the interpretability and broader adoption of remote cognitive measures as accessible and scalable indicators of PD severity. To establish a benchmark, we first analyzed a large in-person dataset (PPMI; *n* = 1,417). We found significant, negative, and small correlations between a cognitive measure (MoCA) scores and two motor measures (MDS-UPDRS III, *ρ* = −0.17; and H&Y scores, *ρ* = −0.12). Second, we administered remote assessments to 152 individuals with PD across 60+ geographical locations, including a videoconference-based MoCA (MoCA-VC) and online versions of the MDS-UPDRS III and H&Y. Consistent with in-person findings, we found significant, negative, and small correlations between the MoCA-VC scores and the two motor measures (MDS-UPDRS III, *ρ* = −0.32; and H&Y scores, *ρ* = −0.16). Additionally, we examined the decrease in the MoCA score as a function of disease stage. A multivariable regression model demonstrated that each one disease stage increase in H&Y was associated with a 0.65-point decrease in MoCA (in-person settings) and a 1.16-point decrease in MoCA-VC (online settings). Together, these findings demonstrate that a remote, home-based cognitive assessment captures motor–cognitive associations comparable to those observed in large in-person settings, supporting its potential utility as an accessible and scalable cognitive index of PD severity.

## Introduction

Parkinson’s disease (PD) is characterized by motor impairments and cognitive deficits. These are routinely assessed to characterize disease severity and treatment needs (Aarsland et al., 2021). Historically, these evaluations have relied almost exclusively on in-person clinical testing (Goetz et al., 2008; Litvan et al., 2012). Yet, despite the importance of these assessments, traditional in-person clinical testing presents several challenges, including limitations in accessibility, scalability, and geographical diversity globally (Binoy et al., 2024a).

Accessibility is a major challenge given that people with PD may experience motor and cognitive impairments that limit their ability to travel to testing sites (Binoy et al., 2023; Saban and Ivry, 2021). Motor impairments, such as postural instability and bradykinesia, can make travel particularly challenging (Moustafa et al., 2016). PD-related fatigue may also contribute to the reduction in capacity to travel to testing sites (Foltynie et al., 2002; Hewitt et al., 2020). Combined, these factors create barriers to in-person participation and clinical-trial enrollment. Scalability poses an additional challenge. In-person assessments are typically administered by expert medical professionals, who are in high demand, leading to long waiting times and increased healthcare costs (Hewitt et al., 2020). This is amplified in remote or non-metropolitan areas, where access to medical centers is limited, thereby reducing demographic and geographical diversity. Accordingly, most studies are conducted within a single institution or restricted geographical region (Binoy et al., 2024a). This may introduce potential sampling bias and limit the generalizability of findings to the broader PD population (Foltynie et al., 2002).

Additionally, although these motor and cognitive assessments are essential, they reflect performance under constrained, clinic-based conditions. Motor and cognitive performance can vary considerably in daily life (Kirk et al., 2025; Regensburger et al., 2025). This can raise concerns about how well clinic-based assessments reflect real-world functioning (Cohen et al., 2023). Understanding motor-cognitive associations in home contexts is therefore increasingly important for both research and clinical use.

Motor severity and disease stage are commonly evaluated using the Movement Disorder Society-Unified Parkinson’s Disease Rating Scale Part III (MDS-UPDRS III; Goetz et al., 2008) and the Hoehn and Yahr (H&Y) scale (Hoehn and Yahr, 1967), respectively. Cognitive status is often assessed with the Montreal Cognitive Assessment (MoCA; Nasreddine et al., 2005), a globally recognized tool used to screen for mild cognitive impairment (MCI; Nasreddine et al., 2005). The MoCA also shows the highest sensitivity for detecting MCI in people with PD (Hoogland et al., 2017). Both tools are commonly used in both clinical care and PD research (Dalrymple-Alford et al., 2010; Hoogland et al., 2017; Hoops et al., 2009; Lima et al., 2012).

Recent work has explored remote or technology-assisted approaches to motor and cognitive testing, including videoconference-based MDS-UPDRS administration (León-García et al., 2023; Monje et al., 2021; Tarolli et al., 2020; Tosin et al., 2024). Multiple studies have employed wearable sensors to track motor symptoms (Adams et al., 2021; Bougea, 2025). In assessing cognition, remote MoCA was found to be feasible and valid in movement disorders, including PD (Binoy et al., 2023; Binoy et al., 2024b; Gilad et al., 2025; De Picciotto et al., 2024).

While these digital methods could improve accessibility and scalability, most studies have examined motor or cognitive performance in isolation, relied on specialized equipment (Adams et al., 2021) or expert raters (León-García et al., 2023). Others included relatively small [e.g., *n* = 25 (Tosin et al., 2024); *n* = 38 (Tarolli et al., 2020); *n* = 39 (León-García et al., 2023)] and geographically restricted samples (one metropolitan city). The extent to which established motor tests relate to cognitive performance in home-based contexts remains to be determined.

One important question is whether remotely collected cognitive performance reflects underlying PD severity. Previous in-person studies have reported small to moderate associations (0.2 < correlation < 0.56) between motor severity and cognitive performance in PD (Gaballa et al., 2025; Gerner et al., 2025; Patrick et al., 2024). Although cognitive impairment is a core feature of PD and is linked to disease severity, existing evidence is derived from in-clinic assessments. While remote cognitive assessments are increasingly used in PD research and clinical monitoring, it remains unclear whether such measures provide an informative and clinically meaningful index of PD severity. Additionally, the extent to which remotely collected cognitive performance reflects disease severity, rather than task-related factors, has not been evaluated. These gaps in literature limit the interpretability and broader adoption of remote cognitive measures for evaluating PD severity in an accessible and scalable manner. Clinical and research studies have increasingly focused on patients in the early to mid-stages PD (Berg et al., 2018); therefore, we chose to focus on this population.

In the current study, we asked whether remote cognitive measures provide an informative index of PD severity. We examined whether motor measures show the expected associations with cognitive performance in early to mid-stage PD when assessed remotely. As a benchmark, we first evaluated these relationships in a large (*n* > 1,000), in-person cohort. We then assessed whether comparable associations are observed when both motor and cognitive evaluations are conducted remotely in patients’ home environments. Motor–cognitive associations were examined in a large (*n* > 100), geographically diverse (60+ locations) PD cohort using the MoCA-VC and online MDS-UPDRS III and H&Y administered via videoconferencing (ZOOM). Next, we examined associations with motor measures separately for motor-dependent and motor-independent MoCA-VC domains. These analyses allow us to examine whether observed motor–cognitive relationships reflect cognitive changes related to disease severity, rather than the motor demands of the cognitive task. Finally, we examined correlations between motor subdomain scores (from the online MDS-UPDRS III) and cognitive subdomains (from the MoCA-VC).

## Methods

### Participants

#### In-person assessment

To provide a benchmark and confirm whether motor–cognitive associations are present in an in-person setting, we analyzed the Parkinson’s Progression Markers Initiative (PPMI) dataset. The study aims and methodology are available on the PPMI website.^1^ We included participants with complete MoCA, MDS-UPDRS III, and H&Y scores. To align with the global goal of examining only early to mid-stage PD, we included participants with H&Y stages 1–3. For comparability with our online sample, when multiple data points were available per participant, we included a randomly selected single visit (excluding screening visits) using simple random sampling. Additionally, MDS-UPDRS III scores were calculated as the total scores without rigidity and postural stability, to align with our online MDS-UPDRS III scores. As an exploratory analysis, we additionally examined cognitive processing speed using the Symbol Digit Modalities Test (SDMT). This test has been suggested to be particularly sensitive to cognitive processing speed deficits in PD (Cao et al., 2024). The final dataset consisted of 1,417 data points.

#### Online assessment

In our online study, 158 participants were recruited from the existing database of the Center for Accessible Neuropsychology (CAN; Algon et al., 2025; Binoy et al., 2023; Binoy et al., 2024b; Gilad et al., 2025; De Picciotto et al., 2024; Saban and Ivry, 2021). For example, participants were recruited through targeted online advertisements, including postings on the Israel Parkinson Association website. Individuals who expressed interest were contacted via email. They were then scheduled for a video-conference session with an experimenter. Inclusion criteria required a confirmed PD diagnosis, fluency in English, Hebrew or Arabic, the ability to provide informed consent, and complete a video-conference session. Remote testing was successfully completed for all participants. Technical difficulties requiring rescheduling occurred in fewer than 5% of cases.

Six participants (3.8%) had H&Y stages 4–5. In order to align with our aim of examining early to mid-stage PD, we excluded these participants, resulting in 152 participants. The study protocol was approved by the Tel Aviv University Ethics Committee.

#### Online assessment procedure

The remote assessments followed the standardized international Protocol for Online Neuropsychological Testing (iPONT; Binoy et al., 2023; Binoy et al., 2024b; Gilad et al., 2025; De Picciotto et al., 2024; Saban and Ivry, 2021). Prior to testing, any video or audio issues were addressed, and sessions were rescheduled if problems could not be resolved within a few minutes. After informed consent, participants completed an online questionnaire that included demographic and medical history information (e.g., age at diagnosis, medication use, primary motor and non-motor symptoms, genetic status, dietary information, and comorbidities). The MoCA-VC (Version 8.1) was then administered, followed by the online version of the MDS-UPDRS III. All assessments were administered and scored by research assistants. The protocol includes a structured, item-by-item guide that raters were required to follow throughout the session (Binoy et al., 2023; Saban and Ivry, 2021).

### MoCA-VC administration

MoCA-VC administration followed the official guidelines outlined on the MoCA website^2^ for remote cognitive assessment and was conducted in accordance with protocols used in previous studies (Binoy et al., 2023; Binoy et al., 2024b; Gilad et al., 2025; Loring et al., 2023; De Picciotto et al., 2024). MoCA-VC was adapted from the standard in-person MoCA (Nasreddine et al., 2005) with modifications appropriate for videoconference administration. Before the session, participants received instructions by email to prepare a blank sheet of paper and a writing utensil. Visuospatial and naming items were presented as individual PowerPoint slides shared on screen. For the Trail Making Test, participants completed the task verbally. The cube-copy and clock-drawing items were displayed on labeled slides (“Copy cube” and “Draw clock”), and participants drew their responses on paper and held them up to the camera for scoring. For the naming task, animal images were presented one at a time, and participants responded verbally. Orientation items were administered by asking participants to close their eyes and report the date and their current location (Binoy et al., 2024b; Saban and Ivry, 2021).

To examine whether the association between MoCA-VC and motor severity measures (online MDS UPDRS III or H&Y) was driven primarily by motor demands, we derived two MoCA-VC subscores. The motor-dependent MoCA-VC subscore was defined as the sum of the Visuospatial/Executive domain (Trails, Cube copy, and Clock drawing; 0–5 points), which requires manual drawing and visuomotor organization. The motor-independent MoCA-VC subscore was defined as the sum of the remaining domains (Naming, Attention, Language, Abstraction, Delayed Recall, Orientation; 0–25 points), which can be performed with minimal motor demands. Finally, we examined the correlations between MoCA-VC subdomains (Visuospatial/Executive, Naming, Attention, Language, Abstraction, Delayed Recall and Orientation) and the motor subdomains derived from the online MDS-UPDRS III to further characterize motor–cognitive associations.

### Online MDS-UPDRS III Administration

Because the evaluations were conducted remotely, we used an abridged version for the online MDS-UPDRS III. The “Rigidity (3.3)” item was omitted because it requires passive movement by the examiner, and the “Postural Stability (3.12)” item was excluded due to the need for a sudden shoulder pull. Prior work has shown that total MDS-UPDRS III scores can be estimated reliably (correlation > 0.95) without these items (Abdolahi et al., 2013; Luo et al., 2022).

Additional adaptations were made to ensure participant safety during videoconference administration. Four items that typically require standing or walking (“arising from chair,” “gait,” “freezing of gait,” and “posture”) were modified so that participants reported their abilities verbally rather than demonstrating them physically. The “kinetic tremor of the hands” item was also adjusted; participants were instructed to touch a nearby object rather than the examiner’s finger during the finger-to-nose task. H&Y staging (range: 1–5) was determined according to standard guidelines based on motor performance (Hoehn and Yahr, 1967) during the videoconference session.

All items were scored using the standard 0–4 scale. With the removal of rigidity and postural stability, the maximum possible score for this scale was 108 (instead of 132). The presence of a caregiver was not required. No adverse events or falls occurred. A detailed, step-by-step description of the protocol is available at this open-access link.^3^

To examine domain-specific motor–cognitive relationships, associations between MoCA-VC cognitive domain scores and online MDS-UPDRS Part III motor subdomain scores were analyzed. Based on previously described frameworks (Goetz et al., 2008; Stebbins et al., 2013), motor subdomain scores were derived to reflect axial/Postural Instability and Gait Disorder (PIGD) features, tremor, and bradykinesia. The axial/PIGD score was defined as the sum of the Speech, Facial Expression, Arising from Chair, Gait, Freezing of Gait, Posture, and Global Spontaneity of Movement items. The tremor score was defined as the sum of Postural Tremor of the Hands, Kinetic Tremor of the Hands, Rest Tremor Amplitude, and Constancy of Rest Tremor items. The bradykinesia score was defined as the sum of Finger Tapping, Hand Movements, Pronation–Supination Movements of the Hands, Toe Tapping, and Leg Agility items.

### Statistical analysis

Data analysis was performed in R using RStudio. All correlations were computed using Spearman’s rank-order correlation (the variables were non-normally distributed and included ordinal measures). Associations were examined between the MoCA scores and the MDS-UPDRS III score or H&Y score for either the In-Person or Online datasets. Outliers in MoCA and MoCA-VC scores were defined as values more than three standard deviations below the mean and were excluded from the H&Y comparison analysis (1.7% of participants in In-Person and 2.0% of participants Online datasets). Finally, differences between dependent correlations (e.g., motor-dependent MoCA-VC–H&Y vs. motor-independent MoCA-VC–H&Y correlations) were tested using Steiger’s *Z* test (Steiger, 1980). We additionally examined associations between MDS-UPDRS III score and H&Y score with SDMT in the In-Person dataset. We further compared the strength of SDMT–motor severity correlations with MoCA–motor severity correlations using Steiger’s *Z* test.

We also utilized linear regression models in both the In-Person and Online datasets. In these models, the MoCA score (MoCA Total/MoCA-VC Total) was the dependent variable. Motor measures (either MDS-UPDRS III or H&Y) were the independent variables. We predicted that individuals with lower MoCA Total scores will have a higher MDS-UPDRS III score (Yin et al., 2025) and a higher H&Y score (Gaballa et al., 2025). In the linear regression models, we also included potential confounded variables: disease duration, age, and medication use (yes/no).

## Results

### Sample characteristics

#### In-person

The total sample was 1,417 participants. The In-Person sample had a mean age of 65.5 years (SE = 0.3), an average of 16.0 years of education (SE = 0.1), and 38.7% women (Table 1). Mean MoCA score was 26.4 (SE = 0.1), mean disease duration was 4.0 years (SE = 0.1), and the average MDS-UPDRS III score was 20.2 (SE = 0.3). All participants were between H&Y stages 1–3.

**Table 1:** Demographic and clinical information.

	*N*	Age (years)	Education (years)	Female (%)	MoCA	Disease duration (years)	MDS-UPDRS III
In-person	1,417	65.5 (0.3) [28–92]	16.0 (0.1) [0–32]	38.7	26.4 (0.1) [6–30]	4.0 (0.1) [0–25]	20.2 (0.3) [2–63]
Online	152	64.8 (0.8) [21–90]	16.5 (0.2) [9–26]	48.7	25.7 (0.3) [10–30]	6.1 (0.4) [0.04–22.1]	20.1 (0.9) [1–62]

Mean (SE) [range].

#### Online

The total sample was 152 participants. Participants were recruited from 60 + distinct geographical locations, most commonly Israel (*n* = 48) and the United States (*n* = 17), demonstrating broad geographical diversity (Table 1). The sample had a mean age of 64.8 years (SE = 0.8), an average of 16.5 years of education (SE = 0.2), and included 48.7% women. Mean MoCA-VC performance was 25.7 (SE = 0.3), mean disease duration was 6.1 years (SE = 0.4), and the average online MDS-UPDRS III score was 20.1 (SE = 0.9). The online MDS-UPDRS III scores did not differ significantly from those of the In-Person cohort (Welch’s *t*-test, *p* = 0.98; Table 1). All participants were between H&Y stages 1–3.

### Relationship between cognitive and motor scores in PD

#### In-person

**Figure 1:**
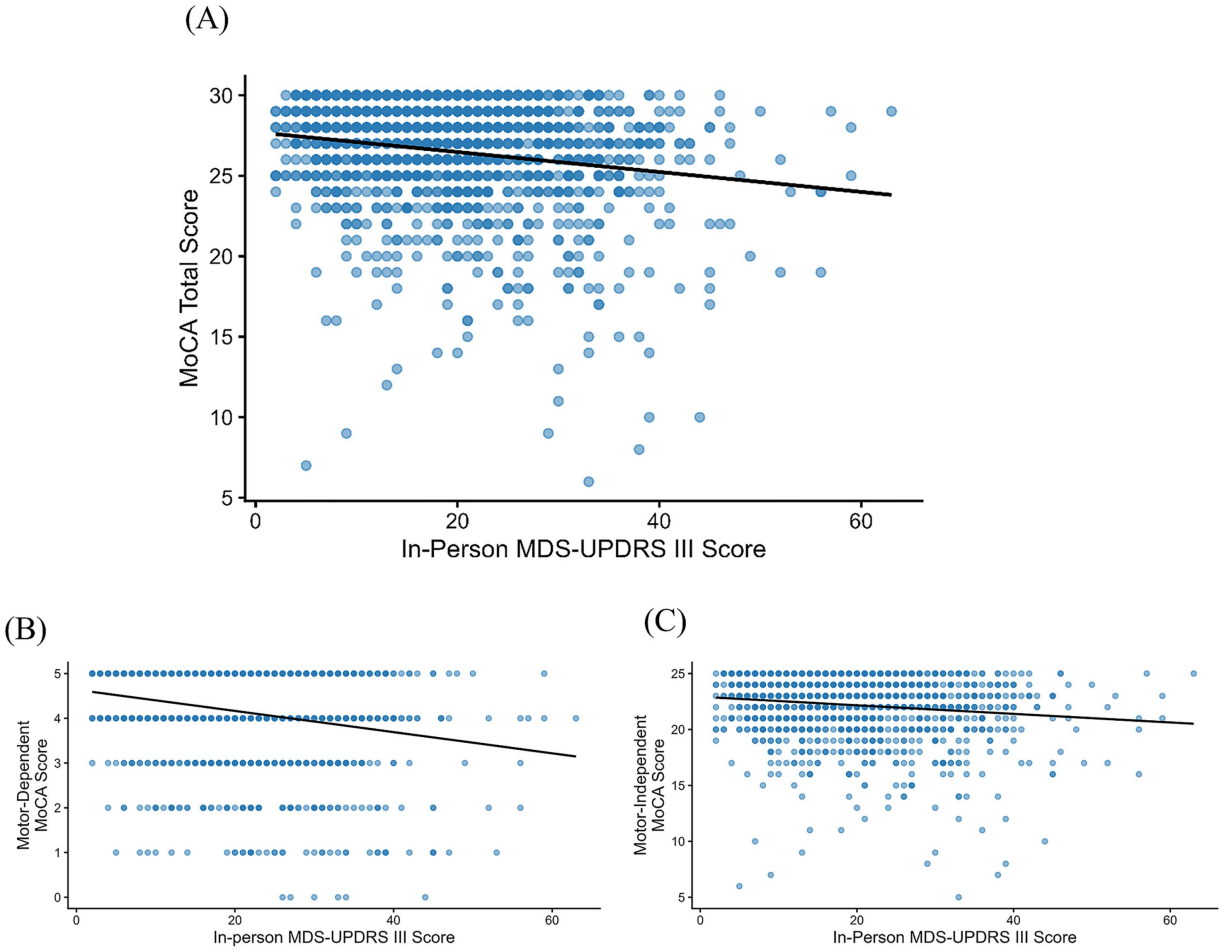
In-person associations between MoCA and MDS-UPDRS III scores. Scatterplots of **(A)** MoCA total score, **(B)** Motor-dependent MoCA subscore, and **(C)** Motor-independent MoCA subscore plotted against in-person MDS-UPDRS III scores. Each point represents one participant. A fitted linear trend line is shown. *N* = 1,417.

We first evaluated correlations in the large-scale (*n* = 1,417) PPMI dataset. MoCA Total scores were significantly and negatively correlated with the MDS-UPDRS III scores (*ρ* = −0.17, *p* < 0.001; Figure 1). A multivariable linear regression model adjusting for disease duration, age, and dopaminergic medication use, indicated that every 10-point increase in the MDS-UPDRS III score was associated with a 0.5-point decrease in MoCA Total score (*β* = −0.05, SE = 0.009, *p* < 0.001). Age was also significantly associated with MoCA Total scores (*β* = −0.07, SE = 0.009, *p* < 0.001). However, disease duration (*β* = 0.01, SE = 0.03, *p* = 0.69) and medication use (*β* = −0.41, SE = 0.22, *p* = 0.058) were not significantly associated with MoCA Total score. The model accounted for 8% of MoCA variability (adjusted *R*^2^ = 0.08).

Both the motor-dependent and motor-independent MoCA subscores were significantly associated with MDS-UPDRS III scores (motor-dependent MoCA: *ρ* = −0.19, *p* < 0.001; motor-independent MoCA: *ρ* = −0.12, *p* < 0.001; Figure 1). The correlation between MDS-UPDRS III scores and the motor-dependent MoCA subscore was significantly stronger than the correlation between MDS-UPDRS III and the motor-independent MoCA subscore (Steiger’s *Z* = 2.28, *p* = 0.022). Notably, both correlations were small (0.12 vs. 0.19).

**Figure 2:**
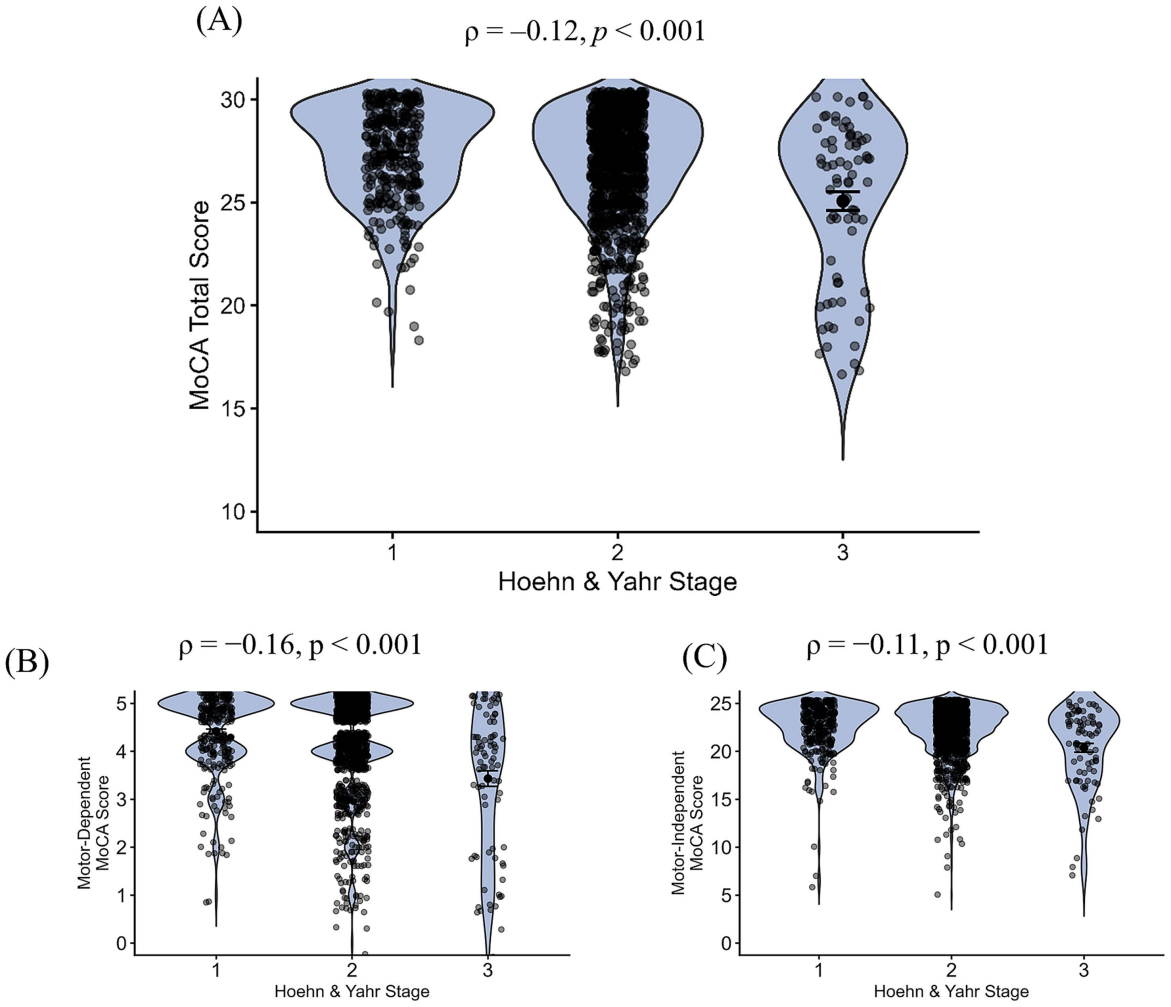
In-person MoCA score as a function of H&Y stage. Violin plot of **(A)** MoCA total score (*N* = 1,392), **(B)** motor-dependent MoCA subscore (*N* = 1,417), and **(C)** motor-independent MoCA subscore (*N* = 1,417) by H&Y stage. Each point represents an individual participant. Violin plots represent kernel-density estimates of the score distributions. Horizontal bars and error bars indicate the group mean and its standard error.

MoCA Total scores were significantly and negatively correlated with H&Y stage (*ρ* = −0.12, *p* < 0.001; Figure 2). A multivariable linear regression model adjusting for disease duration, age, and dopaminergic medication use demonstrated that each one-stage increase in H&Y was associated with a 0.65-point decrease in MoCA (*β* = −0.65, SE = 0.15, *p* < 0.001). Age was also significantly associated with MoCA score (*β* = −0.07, SE = 0.009, *p* < 0.001). Disease duration (*β* = 0.03, SE = 0.03, *p* = 0.30) and medication use (*β* = −0.09, SE = 0.18, *p* = 0.62) were not significantly associated with MoCA scores. The model accounted for 8% of MoCA variability (adjusted *R*^2^ = 0.08).

Both the motor-dependent and motor-independent MoCA subscores were significantly associated with H&Y stage (motor-dependent MoCA: *ρ* = −0.16, *p* < 0.001; motor-independent MoCA: *ρ* = −0.11, *p* < 0.001; Figure 2). There was no significant difference between the strength of these two correlations (Steiger’s *Z* = 1.53, *p* = 0.13). Notably, both correlations were small.

In examining the relation between MoCA subdomains and MDS-UPDRS III subdomains, axial/PIGD and bradykinesia subdomains were associated with lower performance across multiple MoCA cognitive subdomains (Table 2). Axial/PIGD subdomain was significantly correlated with all MoCA subdomains. Bradykinesia was significantly correlated with all MoCA subdomains except for Abstraction. In contrast, tremor severity showed significant correlation with the language subdomain only. The correlation coefficients were small and ranged from 0.06 to −0.22.

**Table 2:** Correlations between the MoCA subdomains and motor subdomains in MDS-UPDRS III in the in-person and online datasets.

Domain	In-person						Online					
	Axial/PIGD		Tremor		Bradykinesia		Axial/PIGD		Tremor		Bradykinesia	
	*ρ*	*p*	*P*	*p*	*P*	*p*	*ρ*	*p*	*ρ*	*P*	*ρ*	*p*
Visuospatial/executive	−0.22	<0.001	−0.03	0.237	−0.17	<0.001	−0.28	<0.001	−0.21	0.011	−0.17	0.041
Naming	−0.05	0.048	−0.03	0.234	−0.06	0.032	−0.04	0.609	−0.22	0.007	−0.06	0.442
Attention	−0.12	<0.001	0.01	0.637	−0.09	0.001	−0.20	0.014	−0.18	0.028	−0.34	<0.001
Recall	−0.09	<0.001	−0.04	0.145	−0.07	0.006	−0.32	<0.001	−0.13	0.109	−0.14	0.076
Language	−0.08	0.002	0.06	0.025	−0.07	0.012	−0.08	0.356	−0.01	0.943	−0.15	0.069
Abstraction	−0.07	0.012	0.00	0.964	−0.04	0.124	−0.12	0.146	−0.06	0.451	−0.06	0.440
Orientation	−0.14	<0.001	−0.03	0.306	−0.10	<0.001	−0.25	0.002	−0.18	0.024	−0.29	<0.001

Spearman’s rho is used to indicate correlation.

Finally, SDMT total scores were significantly associated with the MDS-UPDRS III scores (*ρ* = −0.28, *p* < 0.001). SDMT total scores were also significantly correlated with H&Y stage (*ρ* = −0.26, *p* < 0.001). Interestingly, the association between SDMT and MDS-UPDRS III was significantly stronger than that observed for MoCA Total scores (Steiger’s *Z* = 4.94, *p* < 0.001), indicating MoCA is less sensitive to changes in the MDS-UPDRS III score.

#### Online

**Figure 3:**
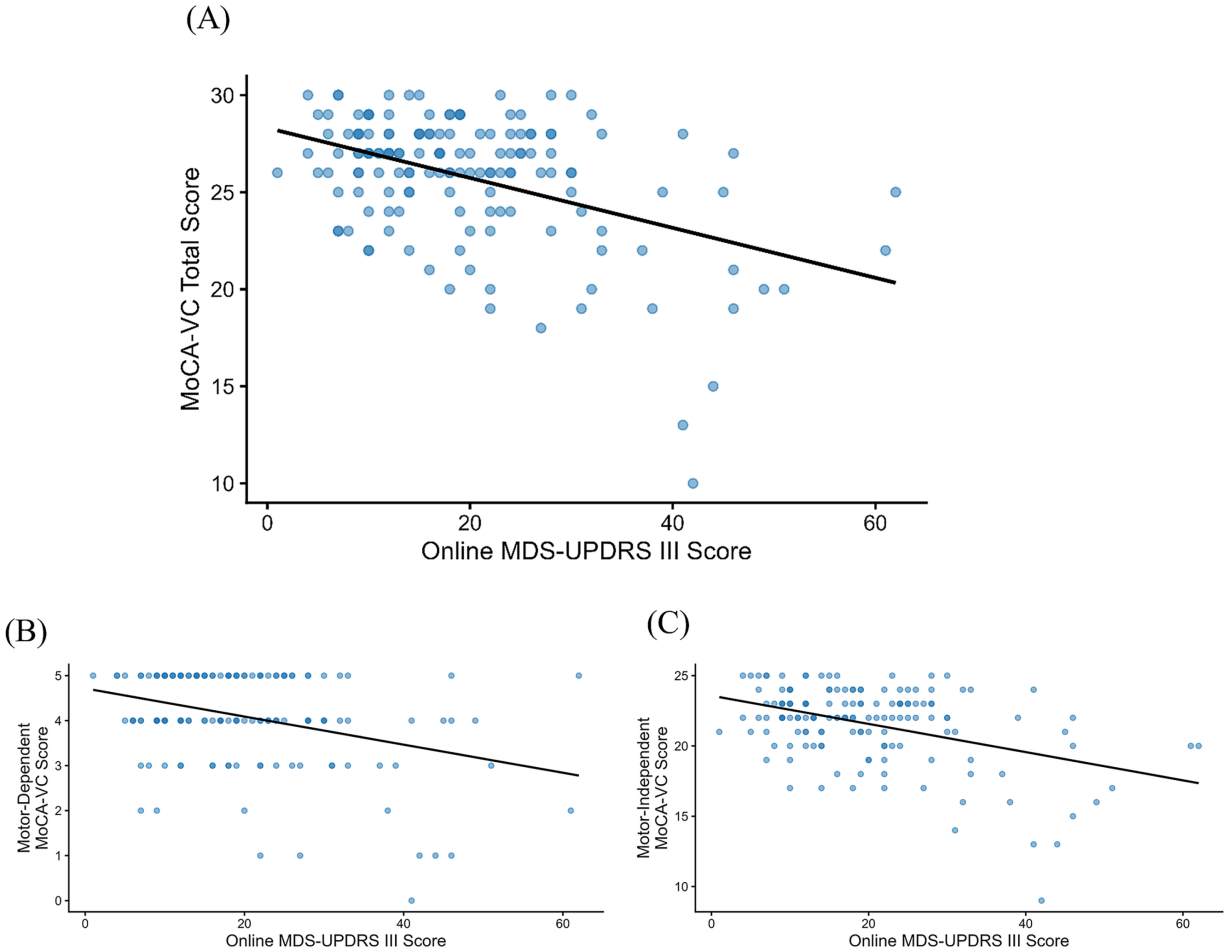
Online associations between MoCA-VC and MDS-UPDRS III scores. Scatterplots of **(A)** MoCA-VC total score, **(B)** motor-dependent MoCA-VC subscore, and **(C)** motor-independent MoCA-VC subscore plotted against online MDS-UPDRS III score. Each point represents one participant. A fitted linear trend line is shown. *N* = 152.

As expected from the analysis of the In-Person dataset, MoCA-VC Total scores were significantly and negatively correlated with the online MDS-UPDRS III scores (*ρ* = −0.32, *p* < 0.001; Figure 3). A multivariable linear regression model adjusting for age, disease duration, and medication use indicated that every 10-point increase in online MDS-UPDRS III score was associated with a 1.4-point decrease in MoCA-VC score (*β* = −0.14, SE = 0.02, *p* < 0.001). Disease duration (*β* = 0.02, SE = 0.05, *p* = 0.63), age (*β* = 0.02, SE = 0.02, *p* = 0.50), and medication use (*β* = 3.22, SE = 1.76, *p* = 0.07) were not significantly associated with MoCA-VC scores. The model accounted for 21% of MoCA-VC variability (adjusted *R*^2^ = 0.21).

Both the motor-dependent and motor-independent MoCA-VC subscores were significantly associated with the online MDS-UPDRS III scores (motor-dependent MoCA-VC: *ρ* = −0.27, *p* < 0.001; motor-independent MoCA-VC: *ρ* = −0.28, *p* < 0.001; Figure 3). There was no significant difference between the strength of these two correlations (Steiger’s *Z* = 0.13, *p* = 0.90), indicating that online MDS-UPDRS III was similarly associated with both subscores of the MoCA-VC.

**Figure 4:**
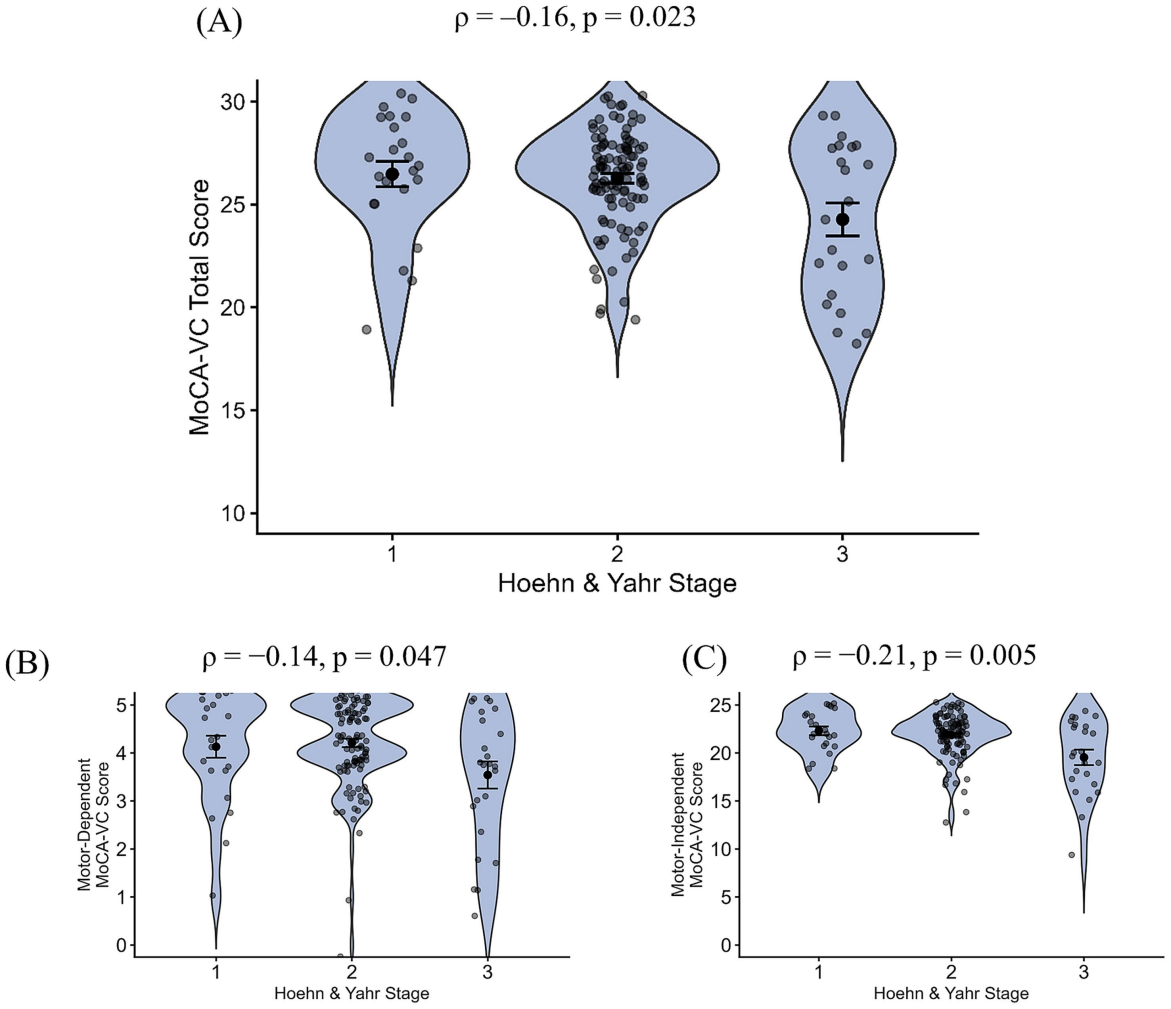
Online MoCA-VC score as a function of H&Y stage. Violin plot of **(A)** MoCA-VC total score (*N* = 149), **(B)** motor-dependent MoCA-VC subscore (*N* = 152), and **(C)** motor-independent MoCA-VC subscore (*N* = 152) by H&Y stage. Each point represents an individual participant. Violin plots represent kernel-density estimates of the score distributions. Horizontal bars and error bars indicate the group mean and its standard error.

As expected, MoCA-VC Total scores significantly declined with increasing H&Y stage (*ρ* = −0.16, *p* = 0.023; Figure 4). A multivariable linear regression model adjusting for age, disease duration, and medication demonstrated that each one-stage increase in H&Y was associated with a 1.16-point decrease in MoCA-VC (*β* = −1.16, SE = 0.48, *p* = 0.003). Disease duration (*β* = 0.02, SE = 0.05, *p* = 0.73), age (*β* = −0.0002, SE = 0.02, *p* = 0.99), and medication use (*β* = 2.70, SE = 1.62, *p* = 0.10) were not significantly associated with MoCA-VC scores. The model accounted for approximately 4% of the variability in MoCA-VC scores (adjusted *R*^2^ = 0.04).

Both the motor-dependent and motor-independent MoCA-VC subscores were significantly associated with H&Y stage (motor-dependent MoCA-VC: *ρ* = −0.14, *p* = 0.047; motor-independent MoCA-VC: *ρ* = −0.21, *p* = 0.005; Figure 4). There was no significant difference between the strength of these two correlations (Steiger’s *Z* = 0.82, *p* = 0.42).

In examining the correlation of subdomains of online MDS-UPDRS III and subdomains of MoCA-VC, the axial/PIGD subdomain was significantly associated with lower performance in Visuospatial/Executive, Attention, Recall and Orientation (Table 2). Bradykinesia was significantly associated with Visuospatial/Executive, Attention and Orientation subdomains of MoCA-VC. The tremor score exhibited significant correlations with Visuospatial/Executive, Naming, Attention and Orientation. The correlation coefficients were small and ranged from −0.01 to −0.34.

## Discussion

In this study, we examined motor–cognitive associations in PD through remote assessments completed in participants’ homes. Across a relatively large and geographically diverse cohort, we found that two motor measures (online MDS-UPDRS III and H&Y) were significantly correlated with cognitive performance (MoCA-VC). Although the correlations were small, they were in the expected direction and are consistent with both our analysis of a large-scale dataset (>1,000) and previous clinic-based studies (Murakami et al., 2013; Patrick et al., 2024). These results indicate that motor–cognitive associations remain detectable even when assessments are performed remotely in home environments.

Previous in-person studies have generally reported small-medium associations between motor severity and cognitive performance in PD. A recent systematic review of MoCA and motor symptoms in PD found an across-studies negative correlation between MoCA scores and motor severity, with a small pooled correlation of *r* ~ −0.22 (Patrick et al., 2024). Associations between MoCA and H&Y stage have also been observed. One study reported a correlation of *r* ~ −0.40 (Gaballa et al., 2025) and another study found a correlation of *r* ~ −0.55 (Gerner et al., 2025).

In addition, both motor-dependent and motor-independent MoCA-VC domains demonstrated significant associations with both motor measures. Furthermore, these correlations did not differ in strength in the online dataset. This suggests that the cognitive differences captured in home-based remote testing reflect cognitive changes rather than task-related motor demands. In contrast, in the In-Person dataset, we found a significant difference such that the motor-dependent cognitive score showed a stronger correlation with disease severity than the motor-independent cognitive score. Given that we did not find significant differences in the online dataset, this pattern of results indicates potential differences between online and in-person assessments that should be further examined in future studies. However, it is worth noting that both correlations were small in the in-person settings (< 0.2).

Five limitations should be noted. First, the participants were in early to mid-stage PD (H&Y 1–3). Although we aimed to focus on this specific subpopulation, the findings may not generalize to later disease stages. Second, the online MDS-UPDRS III necessarily excludes items that cannot be administered safely online, and several balance-related items relied on self-report. Although previous work suggests that these omissions still allow for reasonable estimation of overall motor severity (Abdolahi et al., 2013; Luo et al., 2022; Sangarapillai et al., 2022), additional validation will be important. Third, because this is a cross-sectional study, the observed associations do not permit causal inference. Fourth, they cannot determine whether remote assessments capture within-person change over time. Fifth, our chosen tools (e.g., MoCA and MDS-UPDRS III) do not capture the full range of cognitive or motor abilities. Future studies should incorporate additional measures to more comprehensively assess motor–cognitive associations in PD. In this context, we conducted an exploratory comparison in the In-Person dataset between a global cognitive measure (MoCA) and a processing speed–focused task (SDMT), given prior evidence suggesting heightened sensitivity of SDMT to cognitive impairment in PD (Cao et al., 2024). Such tasks represent promising candidates for future investigation and adaptation within remote assessment frameworks.

Additionally, the association between motor severity and cognitive scores was stronger in the Online dataset than in the In-Person dataset. This difference may reflect a combination of factors. First, the testing settings may contribute to variability; assessments conducted in participants’ homes may capture more typical functioning but are less standardized than clinic-based evaluations. Second, methodological differences in motor assessment, including the self-report in the Online MDS-UPDRS III, may have influenced the strength of the observed associations. Direct within-subject comparisons across in-person and online settings will be necessary to further understand these factors.

One benefit of remote testing is the ability to increase diversity. The PD cohort in this online study included 48.7% women, yielding an approximately equal sex distribution. This contrasts with many large PD studies, which typically report a male predominance of ~60–66% (Jesús et al., 2020; Pavelka et al., 2023; Simuni et al., 2018). Additionally, previous studies were typically with a small sample size (León-García et al., 2023; Monje et al., 2021; Tarolli et al., 2020; Tosin et al., 2024), in a single testing site (Tarolli et al., 2020), or limited geographical diversity (León-García et al., 2023). In contrast, our approach was deployed across 60+ distinct geographical locations in two countries, demonstrating broad geographical diversity. Our assessments were conducted in participants’ homes across 60 + distinct locations, rather than at a single clinical site. The fact that motor–cognitive associations remained detectable under these home conditions highlights the potential value of remote assessment for capturing meaningful indicators of disease severity in the comfort of the patient’s home.

Taken together, the present findings represent an initial step toward addressing several key conceptual and translational challenges in PD research. First, there is clinical heterogeneity in PD. Thus, there is substantial variability in cognitive profiles even within the same H&Y or MDS-UPDRS stages. While the current cross-sectional design was not intended to identify distinct phenotypic subtypes, scalable remote assessments may provide a practical framework for future studies aimed at characterizing inter-individual variability and phenotypic trajectories beyond the traditional motor staging systems. Second, the dopaminergic ON/OFF states and treatment adherence may influence remotely collected outcomes. Although medication state was not experimentally manipulated in the present study, home-based remote assessments may be particularly well suited for capturing symptom fluctuations related to medication timing. This issue is of increasing relevance in aging PD populations and one that is difficult to address using conventional clinic-based evaluations. Third, there are implications for higher-frequency and longitudinal monitoring. The demonstrated feasibility of remote motor and cognitive assessments supports future integration with more frequent, brief, or gamified task paradigms delivered via tablets or consumer devices. Such approaches could enable sensitive tracking of disease progression and intra-individual change over time, which is especially important in early and mid-stage PD. Fourth, certain elements of the current framework could be extended to real-world functioning and activities of daily living. Future work may incorporate complementary passive or semi-passive digital markers (e.g., smartphone-based motor or behavioral signals) to augment structured assessments, modalities that were not evaluated in the present study. Finally, videoconference-based assessments inherently generate audio data. Thus, future studies could examine whether speech and acoustic features extracted from these recordings provide additional insight into motor and cognitive status in PD. This represents a promising extension of the current methodology rather than a conclusion supported by the present data.

The current results should not be viewed with high clinical value yet. However, they provide an initial step that could inform the development of a cognitive index for PD severity. The present findings suggest a promising direction toward the development of an inclusive, scalable cognitive index for PD that can be applied across diverse populations and home-based settings.

## Data Availability

The datasets generated for this study are not publicly available due to patient privacy and ethical restrictions. The data are available from the corresponding author upon reasonable request, subject to approval by the relevant institutional ethics committee and in accordance with applicable data protection regulations.

## Footnotes

1
http://www.ppmi-info.org/study-design

2
https://mocacognition.com/

3
https://docs.google.com/document/d/1n5eQrH63rWN2vRxAVrZ-c2SMt_U0fOCHyJPhYJGjhCA/edit?tab=t.0
